# Downregulation of Cullin 3 Ligase Signaling Pathways Contributes to Hypertension in Preeclampsia

**DOI:** 10.3389/fcvm.2021.654254

**Published:** 2021-04-13

**Authors:** Ya Zhang, Gengru Jiang, Chong Zhang

**Affiliations:** Department of Nephrology, Xin Hua Hospital Affiliated to Shanghai Jiao Tong University School of Medicine, Shanghai, China

**Keywords:** preeclampsia, CRL3, Jab1/CSN5, hypoxia-inducible factor 1α, peroxisome proliferator-activated receptor gamma

## Abstract

**Background:** Preeclampsia (PE) is a leading cause of maternal and perinatal morbidity and mortality; however, its etiology and pathophysiology remain obscure. PE is initiated by inadequate spiral artery remodeling and subsequent placental ischemia/hypoxia, which stimulates release of bioactive factors into maternal circulation, leading to hypertension and renal damage.

**Methods and Results:** Abundance of key components of cullin 3-ring ubiquitin ligase (CRL3), including cullin 3 (CUL3) and its neddylated modification, and adaptors including Kelch-like 2 (KLHL2) and Rho-related BTB domain containing protein 1 was all decreased in spiral arteries and placentas of PE patients. Similar changes were found in aortic tissues and renal distal tubules of pregnant mice treated with Nω-nitro-l-arginine methyl ester hydrochloride. The downregulation of CRL3 function led to accumulation of with-no-lysine kinases, phosphodiesterase 5, and RhoA in vessels and renal distal tubules, which promoted vasoconstriction and Na–Cl cotransporter activation in the distal convoluted tubule (DCT), as well as vascular and DCT structure remodeling. Proton pump inhibitor esomeprazole partially restored CRL3 function. *In vitro* studies have shown that increased abundance of JAB1, a component of the COP9 signalosome, inhibited CUL3 neddylation and promoted the expression of hypoxia-inducible factor 1α, which downregulated peroxisome proliferator–activated receptor γ and further promoted CUL3 inactivation. KLHL3/2 was degraded by increased autophagy.

**Conclusion:** These findings support that the downregulation of CRL3 function disrupts the balance of vasoconstriction and vasodilation and aggravates excess reabsorption of sodium in PE.

## Introduction

Preeclampsia (PE) is a pregnancy-specific hypertensive disorder and the leading cause of maternal and fetal morbidity and mortality affecting 6 to 10% of hypertensive pregnancies (1, 2). Although prevalent, the pathophysiology of PE is obscure. Up to now, the pathogenesis and development of PE have been divided into several steps, including decreased trophoblast invasion, impaired spiral artery remodeling, placental ischemia/hypoxia, and circulating bioactive factors (3, 4). Importantly, spiral arteries must undergo remodeling under normal pregnancy, indicating that vascular smooth muscle (VSM) should be replaced by fibrinoid material (5). Transformation to high-capacity, low-resistance vessels is important for adequate fetal blood and oxygen supply. Failure of this process by VSM retention results in placental ischemia/hypoxia and abnormal release of circulating bioactive factors (6). It can cause disorders in maternal cardiovascular, renal, and cerebral systems, and patients exhibit hypertension, proteinuria, and seizures. However, the effective treatment of PE is lacking; the only effective treatment includes termination of pregnancy.

Recent studies have demonstrated that silencing cullin 3 (CUL3) in the trophoblast cells leads to decreased trophoblast invasion and may contribute to the onset of PE (7). CUL3 is a molecular scaffold of cullin 3-ring ubiquitin ligase (CRL3), which can be activated by covalent binding to NEDD8 (neuronal precursor cell expressed developmentally downregulated protein 8), a process called neddylation (8). The COP9 signalosome (CSN) complex can remove NEDD8 from CUL3 through its deNEDDylase activity. CSN5, also known as JAB1, is the key CSN subunit that catalyzes the deNEDDylase function (9). Supporting this theory, Cayli et al. reported that CSN5 expression in human pre-eclamptic placentas was significantly increased (10).

CUL3, with the help of its various substrate recognition proteins, also known as adaptors containing BTB domains, recognizes specific substrates for ubiquitination and then proteasomal degradation. A reduction in CUL3 expression in VSM causes abnormal vasoconstriction and hence hypertension (11). Rho-related BTB domain containing protein 1 (RhoBTB1) serves as adaptor of CUL3 for degradation of phosphodiesterase 5 (PDE5), which promotes the cyclic 3′,5′-monophosphate–dependent relaxation of VSMCs (12). Moreover, conditional genetic ablation of CUL3 causes the accumulation of RhoA, a small GTPase modulating Rho kinase (ROCK) activity in VSM, and promotes vasoconstriction (11, 13, 14). Recently, Zeniya et al. reported that Kelch-like 2 (KLHL2), a Kelch-like family member expressed in VSM, mediates ubiquitination of With-No-lysine(K) kinase 3 (WNK3) for CRL3 degradation (15). WNK3–OSR1/STE20/SPS1-related proline/alanine-rich kinase (SPAK/OSR1)–Na/K/Cl cotransporter isoform 1 (NKCC1) signal in mouse arteries has been shown to participate in the regulation of VSM contraction and blood pressure.

Clinical studies have shown that PE patients exhibit sodium retention compared to healthy pregnant patients (16, 17), suggesting a possible role of renal tubule dysfunction on the development of hypertension in PE. CRL3 plays a key role in regulating blood pressure not only through its effect on vasculature, but also through its effect on the distal renal tubule. Kelch-like 3 (KLHL3) serves as a substrate-binding adaptor of CUL3 in the distal convoluted tubule (DCT) for proteasomal degradation of their substrates including WNK4 and WNK1 (18). Disease-causing mutations in any of these four genes cause an inherited disease named familial hyperkalemic hypertension (FHHt, also called pseudohypoaldosteronism type II), which is characterized by hypertension, hyperkalemia, and metabolic acidosis (19). Elevated protein levels of WNK4 and/or WNK1 in turn phosphorylate and activate SPAK/OSR1 and thiazide-sensitive Na–Cl cotransporter (NCC; encoded by *SLC12A3*), which account for the phenotype of FHHt (20).

In this study, we investigated the implications of CRL3 on blood pressure in pregnant women with PE, as well as pregnant mice with hypertension while illuminating a possible mechanism for CRL3 dysfunction in PE.

## Methods

### Tissue Collection

Ethical approval was obtained for this study from the Human Research Ethics Committee of Xin Hua Hospital Affiliated to Shanghai Jiao Tong University School of Medicine. All participants were given written informed consent before they were included in the study. Placentas were obtained from PE and healthy pregnant women, respectively. For PE patients, no hypertension was found before pregnancy, and pregnancy blood pressure was >140/90 mm Hg with proteinuria of >300 mg/24 h (21), whereas for healthy controls, pregnancy blood pressure was <140/90 mm Hg without other complications. Baseline characteristics, such as maternal age, medical history, use of mediation, and gestational age at delivery, were similarly matched. All women underwent elective cesarean section. All of the tissues were collected within 30 min following delivery and washed in phosphate-buffered saline (PBS). Then, placentas were carefully isolated to extract protein for further analysis.

### Animal Models and Tissue Collection

Animal experiments were performed in accordance with the guidelines set by the Animal Care and Use Committee of Xin Hua Hospital Affiliated to Shanghai Jiao Tong University School of Medicine (no. XHEC-F-2020-004). All male and female wild-type (C57BL/6J) mice were purchased from the Mode I Animal Research Center of Xin Hua Hospital (SYXK 2018-0038). All mice were housed in ventilated cages with up to five mice and a diurnal light cycle providing 12 h of light. Virgin female mice (8–10 weeks old) were placed with a male overnight, and detection of a plug was set as embryonic day (E) 0.5. Pregnant mice were given Nω-nitro-l-arginine methyl ester hydrochloride (l-NAME; MedChemExpress) 50 mg/kg per day in drinking water from E8.5 to E18.5. The plugged females were randomly divided into three groups: control group (*n* = 10), l-NAME group (*n* = 10), and pregnant l-NAME–treated mice with proton pump inhibitor (PPI) (esomeprazole, *n* = 10; Sigma-Aldrich). Pregnant l-NAME–treated mice were given 150 μg of esomeprazole by intraperitoneal injection from E8.5 to E18.5 daily (22). The tail artery systolic pressure was measured with the non-invasive indirect tail-cuff method at E8.5 and E18.5. Pregnant mice were sacrificed at E18.5, and vessels and kidney were collected for further Western blot and morphologic analysis.

### Western Blot Analysis

Kidneys and aortic tissues were extracted and homogenized in lysis buffer (Sangon). Then, they were collected by centrifugation at 4°C for 10 min, and supernatants were collected for analysis of protein concentration by BCA Protein Assay Kit (Thermo Fisher). Total protein was added onto sodium dodecyl sulfate–polyacrylamide gel electrophoresis gel (Sangon), and proteins were then transferred onto polyvinylidene fluoride membranes (Thermo Fisher). After blocking, the membranes were incubated with primary antibodies at 4°C overnight, respectively (Supplementary Table 1). After washing, membranes were incubated with secondary horseradish peroxidase–conjugated antibodies (Cell Signaling Technology, 1:2,000) and then detected by ECL Substrates (Millipore).

### Immunofluorescence Staining

Kidneys were dissected and fixed in 4% paraformaldehyde in PBS and then be rinsed with PBS several times. Mouse kidney sections were collected and boiled in citrate buffer (pH 6.0) for antigen retrieval. After blocking, these sections were incubated with primary antibodies at 4°C overnight, respectively (Supplementary Table 1). Fluorescent dye–conjugated secondary antibodies (Jackson, 1:500) were applied for detection. Then, sections were incubated with DAPI (Sangon) for 5 min. Moreover, after paraffin embedding and slicing, other kidney sections and vascular sections were stained using hematoxylin–eosin (HE) and Masson trichrome staining. Samples were imaged with the microscope (Olympus). Moreover, 10–15 randomly chosen incubated cells were collected from each group. To better visualize the colocalization signal between KLHL3/KLHL2 and specific marker, Pearson correlation coefficient (*R*) and overlap coefficient (*r*) were then calculated. Samples were imaged with the microscope (Leica) and analyzed using the National Institutes of Health ImageJ software.

### Real-Time Quantitative Reverse Transcriptase–Polymerase Chain Reaction

Total RNA was extracted from kidney and aortic tissues from control group and l-NAME group. Molecules, including KLHL3, KLHL2, and CUL3, were detected to verify whether RNA levels of CRL3 were changed after l-NAME administration by real-time quantitative reverse transcriptase–polymerase chain reaction. Six tissues of each group were used for each analysis. Primers are listed: KLHL3-F (ACATCTCATAAATAAGGTGCGAGA), KLHL3-R (CAGTGCATCACCCGCTCTAT), KLHL2-F (TTCCACGCCATGTTTACAGGT), KLHL2-R (AACGTAATCGACCAGCATCC), CUL3-F (ACATCTCATAAATAAGGTGCGAGA), and CUL3-R (CAGTGCATCACCCGCTCTAT).

### Cell Culture

Human embryonic kidney cells (HEK293) and mouse aortic VSM cells (MOVAS) (ATCC) were seeded at 60–70% confluence in Dulbecco modified eagle medium (DMEM) (Hyclone), supplemented with 10% fetal bovine serum (Hyclone) and 1% penicillin/streptomycin (Hyclone). Transfections of WNK4, KLHL3, and JAB1 plasmid (preserved in our laboratory) and JAB1 siRNA (GenePharma) were carried out using Lipofectamine 2000 (Thermo Fisher) and OptiMEM (Thermo Fisher) following the manufacturer's instructions. After transfection for 24 h, cells were incubated at 5% CO_2_ level with 1% O_2_ for 16 h. Meanwhile, we used 100 nM bafilomycin A1 (MedChemExpress) in the DMEM and then collected cells for further Western blot and immunofluorescence (IF), respectively.

### Statistics

Numerical data are presented as means ± SEM. Statistical analysis was performed using GraphPad Prism. An unpaired two-tailed *t*-test was used to determine differences between two groups. An analysis of variance (ANOVA) with Tukey *post hoc* analysis as indicated was used for multiple comparisons. The significance level was set at *p* < 0.05.

## Results

### Downregulation of CRL3 Function and the Accumulation of WNK Kinases, PDE5, and RhoA in Placentas and Spiral Arteries of PE Patients

**Figure 1:**
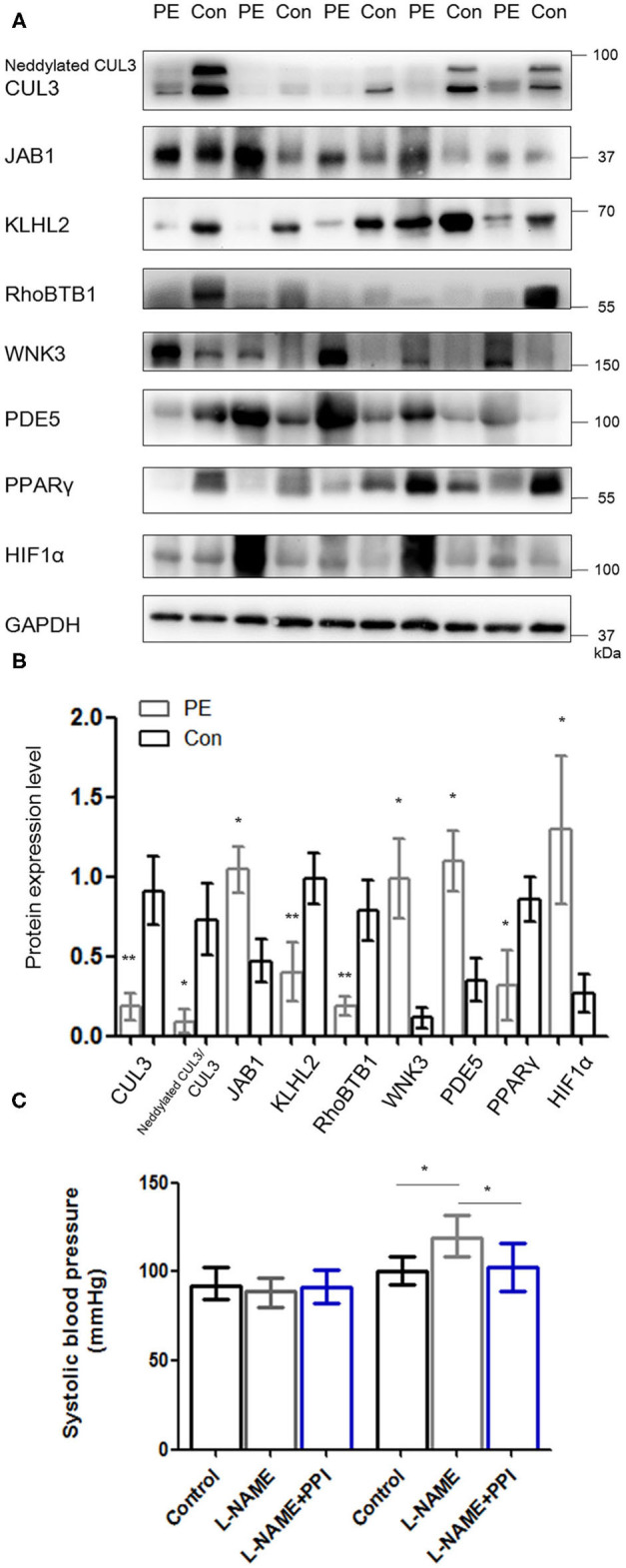
Downregulation of CRL3 function with accumulation of WNK kinases and PDE5 was detected in the placentas of PE patients. **(A**, **B)** Western blotting was performed in the placentas of PE patients and healthy pregnancies, including (1) CUL3 and the ratio of neddylated CUL3 to deneddylated CUL3, (2) the member of the COP9 signalosome JAB1, (3) the adaptors of CRL3 including KLHL2 and RhoBTB1 and their specific substrates including WNK3 and PDE5, and (4) cardioprotective molecule PPARγ and hypoxia-inducible factor 1α (HIF1α). Expressions of these proteins in the placentas were all statistically significant (*t*-test) after normalization to GAPDH between PE and control group. PE indicates preeclampsia (*n* = 5), and Con indicates control (*n* = 5). Data are presented as the mean ± SEM. *p < 0.05 and **p < 0.01. **(C)** Systolic blood pressure were detected among control, l-NAME, and l-NAME + PPI group at E8.5 and E18.5. Data are presented as the mean ± SEM. ANOVA with Tukey *post hoc* analysis, *p < 0.05.

Placentas and spiral arteries from PE patients and normotensive pregnant women were collected for immunoblotting. As shown in Figure 1 and Supplementary Figure 1, the abundance of CUL3 and the ratio of neddylated CUL3 to deneddylated CUL3 were significantly decreased in placentas (*p* < 0.01 and *p* < 0.05, respectively) and spiral arteries (*p* < 0.01 and *p* < 0.05, respectively) in the PE group, whereas JAB1 expression was increased in the placentas (*p* < 0.05) and spiral arteries (*p* < 0.05). Next, we detected the abundance of KLHL2 and RhoBTB1, both of which serve as adaptor proteins of CRL3 in vasculature. Immunoblots revealed substantially decreased abundance of both KLHL2 and RhoBTB1 in placentas (*p* < 0.01 and *p* < 0.05, respectively) and spiral arteries (*p* < 0.01 and *p* < 0.01, respectively) of the PE group. Accumulation of WNK3 and PDE5 in placentas (*p* < 0.05 and *p* < 0.05, respectively) and spiral arteries (*p* < 0.05 and *p* < 0.05, respectively) with PE was detected, which was accompanied by downregulation of CUL3 and its adaptors. Peroxisome proliferator–activated receptor γ (PPARγ) is widely expressed in the VSM of aortic tissues and placenta (23). PPARγ exerts its cardioprotective effect and promotes vascular dilation via different target genes including CUL3 and its adaptors in VSM. PPARγ expression was significantly reduced both in the placentas and spiral arteries in PE group (*p* < 0.05 and *p* < 0.05, respectively). JAB1 plays a key role in maintaining the stability of hypoxia-inducible factor 1α (HIF1α) *in vitro*, especially under hypoxic conditions (24). HIF1α expression was substantially increased in placentas of the PE group (*p* < 0.05), which further supported that placentas were suffering from ischemia/hypoxia. Abundance of α-smooth muscle actin (α-SMA) was significantly increased in the PE spiral arteries (*p* < 0.01) (Supplementary Figure 1), showing that VSM failed to be replaced by fibrinoid material. Furthermore, spiral artery remodeling was inadequate, consistent with previous research (25). Abundance of RhoA was also significantly increased in the spiral arteries of PE patients (*p* < 0.01). These findings support our hypothesis that the downregulation of CRL3 function participates in the pathogenesis and development of PE. Consequently, upregulation of WNK kinases and PDE5 in VSM enhanced vasoconstriction and impaired placental blood flow, which promoted release of circulating bioactive factors from placenta into maternal circulation.

### Downregulation of CRL3 Function and the Accumulation of WNK Kinases, PDE5, and RhoA/ROCK Activity in Aortic Tissues of Pregnant l-NAME–Treated Mice

To investigate whether the activity of CRL3 is involved in the molecular mechanisms of maternal hypertension and renal damage in PE, pregnant mice were administered with l-NAME in drinking water beginning on E8.5 and were euthanized at E18.5 (26). Significantly elevated blood pressure was shown in l-NAME group at E18.5 (*p* < 0.05) (Figure 1C). Urinalysis strips indicated that l-NAME mice exhibited obvious proteinuria (++~+++), and the control group showed absent to mild proteinuria.

**Figure 2:**
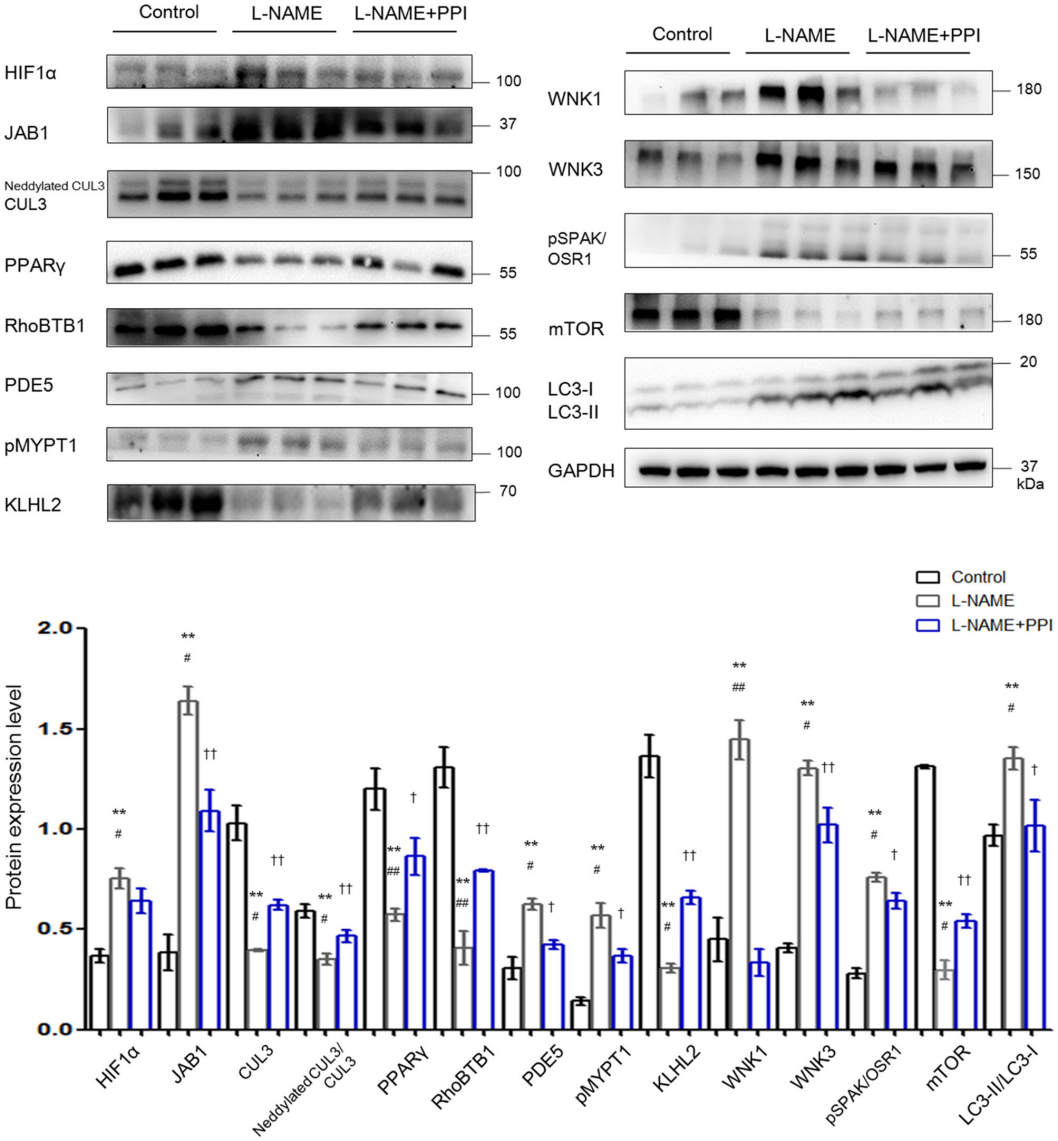
Downregulation of CRL3 function caused accumulation of PDE5 and WNK kinases and increased RhoA/ROCK activity in the aortic tissues of pregnant l-NAME–treated mice. Protein levels were detected by Western blotting, including (1) hypoxia-inducible factor 1α (HIF1α), member of the COP9 signalosome JAB1, CUL3 abundance and neddylation, and cardioprotective molecule PPARγ; (2) adaptor protein RhoBTB1 and its substrate PDE5 and the index of RhoA/ROCK activity phosphorylated MYPT1; (3) adaptor protein KLHL2 and its substrates including WNK1 and WNK3 and downstream phosphorylated SPAK/OSR1; and (4) autophagy markers mTOR and LC3-II/LC3-I. Expressions of these proteins in the aortic tissues were all statistically significant after normalization to GAPDH among control, l-NAME, and l-NAME + PPI group (ANOVA with Tukey *post hoc* analysis). Data are presented as the mean ± SEM. **p < 0.01 compared between control and l-NAME group. ^#^*p* < 0.05 and ^##^*p* < 0.01 compared between l-NAME and l-NAME + PPI group. †*p* < 0.05 and ††*p* < 0.01 compared between control and l-NAME + PPI group.

Previous studies have reported that elevated abundance of HIF1α is related to preeclamptic symptoms, and its level is increased not only in the placentas but also in circulation (3, 27). Figure 2 demonstrates HIF1α abundance was indeed increased in the aortic tissues of PE mice (*p* < 0.01). Furthermore, JAB1 expression was significantly increased (*p* < 0.01), whereas abundance and neddylation of CUL3 were decreased in the aortic tissues of l-NAME group (*p* < 0.01 and *p* < 0.01, respectively). These data show the balance between neddylation and deneddylation was disturbed in mice with PE. Recent studies have demonstrated that RhoBTB1 in aortic tissues is modulated by PPARγ, which promotes vasodilation by increasing RhoBTB1 abundance (12). As shown in Figure 2, the abundance of PPARγ and RhoBTB1 was both substantially decreased in l-NAME group (*p* < 0.01 and *p* < 0.01, respectively). To address the alterations of vasodilation and vessel wall contractility, we detected the level of PDE5 and phosphorylated myosin light chain phosphatase subunit (pMYPT1), an index of RhoA/ROCK activity, and revealed their protein levels were both significantly increased (*p* < 0.01 and *p* < 0.01, respectively). WNK3 and WNK1, which was degraded by CUL3 and KLHL2, also participate in modulating the constriction and relaxation of aortic tissues. KLHL2 expression was reduced (*p* < 0.01), and the corresponding substrates WNK3 and WNK1 were both substantially increased (*p* < 0.01 and *p* < 0.01, respectively). Additionally, phosphorylated SPAK/OSR1 (pSPAK/OSR1) was also increased in l-NAME group (*p* < 0.01). As KLHL3 and KLHL2 can be decreased by autophagy-mediated degradation and proteasomal degradation (28), we detected the expression of autophagy markers isoform of light chain 3 (LC3) and mammalian target of rapamycin (mTOR). Abundance of mTOR was significantly decreased, and LC3-II/LC3-I abundance was increased in the l-NAME group (*p* < 0.01 and *p* < 0.01, respectively). These results suggested that l-NAME administration increased the autophagy of KLHL2 in aortic tissues.

Epidemiological studies have identified that PPI could be considered as potential effective therapeutics for PE (22). PE patients who received PPI exhibited less gestational hypertension compared to PE patients without PPI. Similarly, our results demonstrate that after administration of PPI, pregnant l-NAME–treated mice have reduced blood pressure and HIF1α expression in the maternal vessels (*p* < 0.05 and *p* < 0.05, respectively). PPI administration also partially decreased the degradation of RhoBTB1 and accumulation of PDE5 in the aortic tissues of l-NAME group (*p* < 0.01 and *p* < 0.05, respectively). Additionally, PPI treatment modestly increased the protein levels of KLHL2 and CUL3, as well as CUL3 neddylation in aortic tissues of l-NAME mice (*p* < 0.05, *p* < 0.05, and *p* < 0.05, respectively). Decreased accumulation of WNK3 and WNK1 in vessels was found in the aortic tissues of l-NAME–treated mice with PPI treatment (*p* < 0.05 and *p* < 0.01, respectively).

Increased medial wall thickness and proliferation of VSM cells were considered to be associated with the pathogenesis of hypertension (14). We observed morphometric changes in the vasculature under PE by HE, Masson, and IF staining. Morphometric analysis indicated that there was an increase in aortic tissue thickness in the l-NAME group (*p* < 0.01) (Supplementary Figure 2). Similar to aortic tissues, there was an increase in thickness of renal vessels in pregnant l-NAME–treated mice; α-SMA expression was increased in the kidney, similar to previous reports, which demonstrated relatively lower maternal renal blood flow in PE (4, 29) (Supplementary Figure 2). Esomeprazole treatment partially ameliorated the thickening of vessels in the l-NAME group.

### Downregulation of CRL3 Function and the Accumulation of WNK Kinases in the Kidney of Pregnant l-NAME–Treated Mice

**Figure 3:**
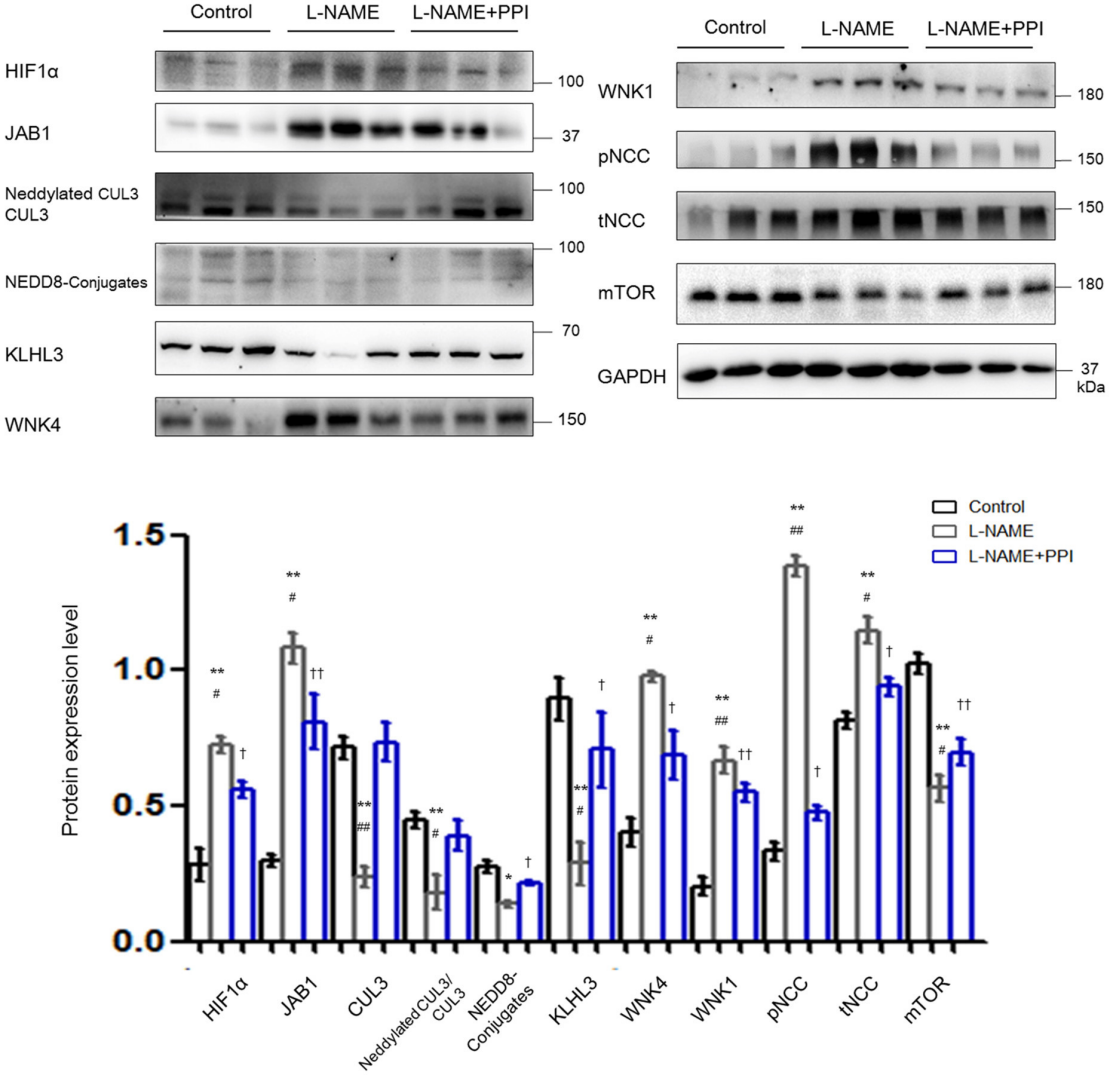
Downregulation of CRL3 function was involved in the accumulation of WNK kinases in the kidney of pregnant l-NAME–treated mice. Protein levels were detected by Western blotting, including (1) hypoxia-inducible factor 1α (HIF1α), member of the COP9 signalosome JAB1, CUL3 abundance and neddylation, NEDD8 conjugates and adaptor KLHL3; (2) substrates of CRL3 including WNK4 and WNK1 and phosphorylated NCC (pNCC) and total NCC (tNCC); and (3) autophagy marker mTOR. Expressions of these proteins in the kidney were statistically all significant after normalization to GAPDH among control, l-NAME, and l-NAME + PPI group (ANOVA with Tukey *post hoc* analysis). Data are presented as the mean ± SEM. *p < 0.05 and **p < 0.01 compared between control and l-NAME group. ^#^*p* < 0.05 and ^*##*^*p* < 0.01 compared between l-NAME and l-NAME + PPI group. †*p* < 0.05 and ††*p* < 0.01 compared between control and l-NAME + PPI group.

As shown in Figure 3, HIF1α abundance was significantly increased in kidney after l-NAME treatment when compared with untreated pregnant mice (*p* < 0.01). Similar to the changes detected in the aorta, JAB1 expression was increased (*p* < 0.01), and abundance and neddylation of CUL3 were both significantly reduced in the kidney of the l-NAME group (*p* < 0.01 and *p* < 0.01, respectively). Meanwhile, the abundance of total NEDD8 conjugates and the adaptor KLHL3 expression were also substantially decreased (*p* < 0.05 and *p* < 0.01, respectively). Downregulation of CRL3 function, indicated by decreased KLHL3 and CUL3 abundance and neddylation, was accompanied by the accumulation of substrates, including WNK4 and WNK1 (*p* < 0.01 and *p* < 0.01, respectively). Phosphorylation of downstream SPAK/OSR1 was increased. Both abundance of total NCC and that of phosphorylated NCC were substantially increased in pregnant l-NAME–treated mice (*p* < 0.01 and *p* < 0.01, respectively). Abundance of mTOR was significantly decreased (*p* < 0.01) in the kidney after l-NAME administration. However, total mRNA of KLHL3/KLHL2 and CUL3 showed no significant difference in the kidney and aortic tissues (Supplementary Figure 2) between the control and l-NAME groups, suggesting a posttranslational regulation of CUL3 and KLHL3/2.

**Figure 4:**
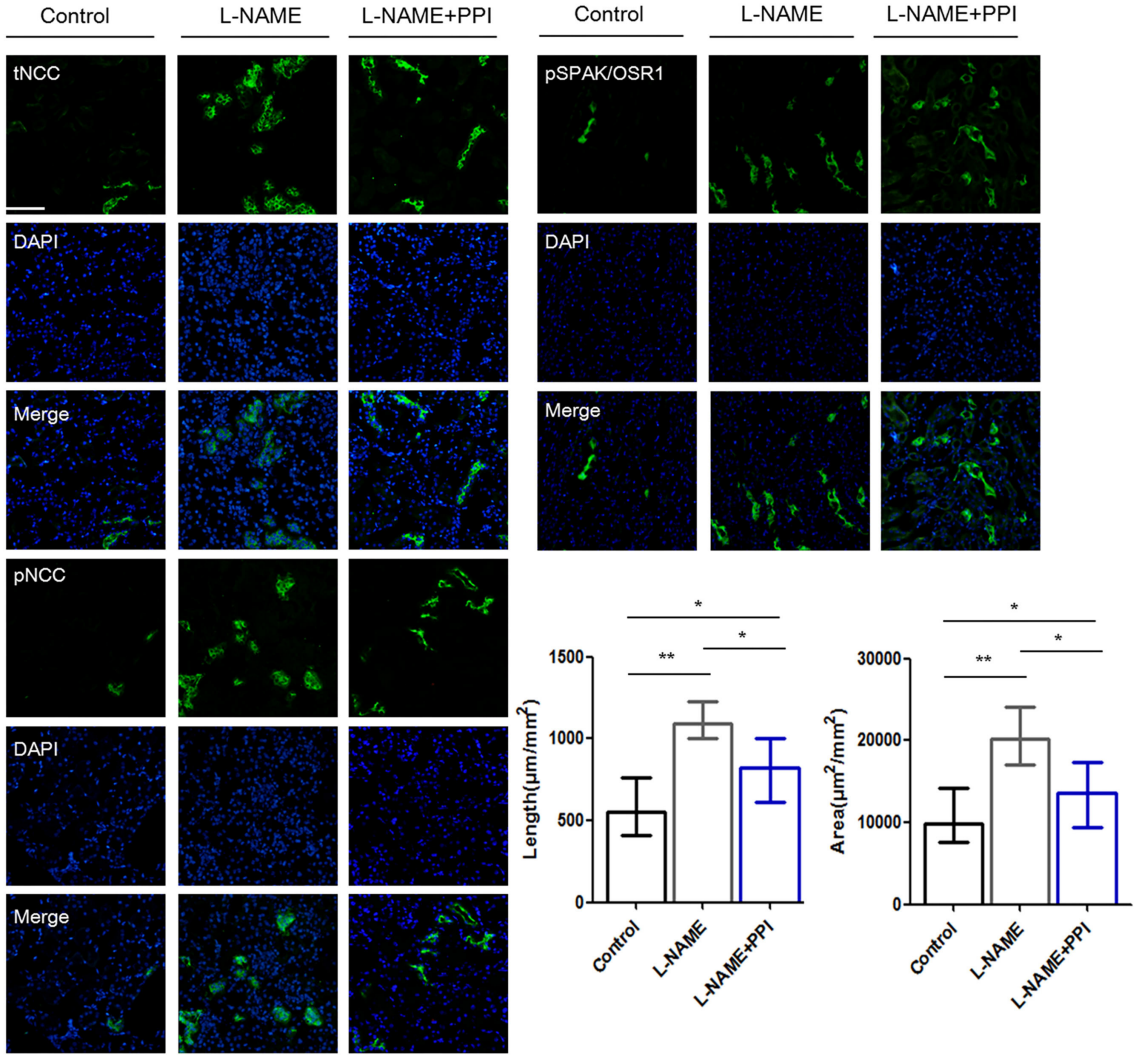
Distal renal tubule remodeling of pregnant l-NAME–treated mice. Representative immunofluorescence images including total NCC (tNCC), phosphorylated NCC (pNCC), and phosphorylated SPAK/OSR1 (pSPAK/OSR1) staining images of the distal renal tubules. Scale bars: 100 μm. Histological analysis of the length and area expressing tNCC in the distal renal tubule among control (*n* = 6), l-NAME (*n* = 6), and l-NAME + PPI group (*n* = 6) (ANOVA with Tukey *post hoc* analysis). Data are presented as the mean ± SEM (*p < 0.05 and **p < 0.01).

Distal tubule was then further evaluated by IF staining and imaging in pregnant l-NAME–treated mice (Figure 4). Consistently, NCC expression and its phosphorylation were both significantly increased in the l-NAME group (*p* < 0.01 and *p* < 0.01, respectively). It was reported that constitutive NCC activation increases the length of the DCT, whereas NCC inhibition shortens it (30). Therefore, the length of identifiable distal renal tubule expressing NCC was measured and normalized to the area of the cortex in the image (μm/mm^2^) (30). The length and area of distal tubules expressing NCC were both significantly increased after l-NAME administration (*p* < 0.01 and *p* < 0.01, respectively), and these changes could be partially prevented by esomeprazole treatment (*p* < 0.05 and *p* < 0.05, respectively). Thus, our study suggests that l-NAME administration leads to DCT remodeling in pregnant mice, which further demonstrates that NCC activity is abnormally activated in PE. Hence, downregulation of CRL3 function promotes DCT remodeling in pregnant l-NAME–treated mice, which results in the development of severe hypertension in PE.

### Downregulation of CRL3 Function and the Activation of SPAK/OSR1 in HEK293 Cells Under Hypoxia

**Figure 5:**
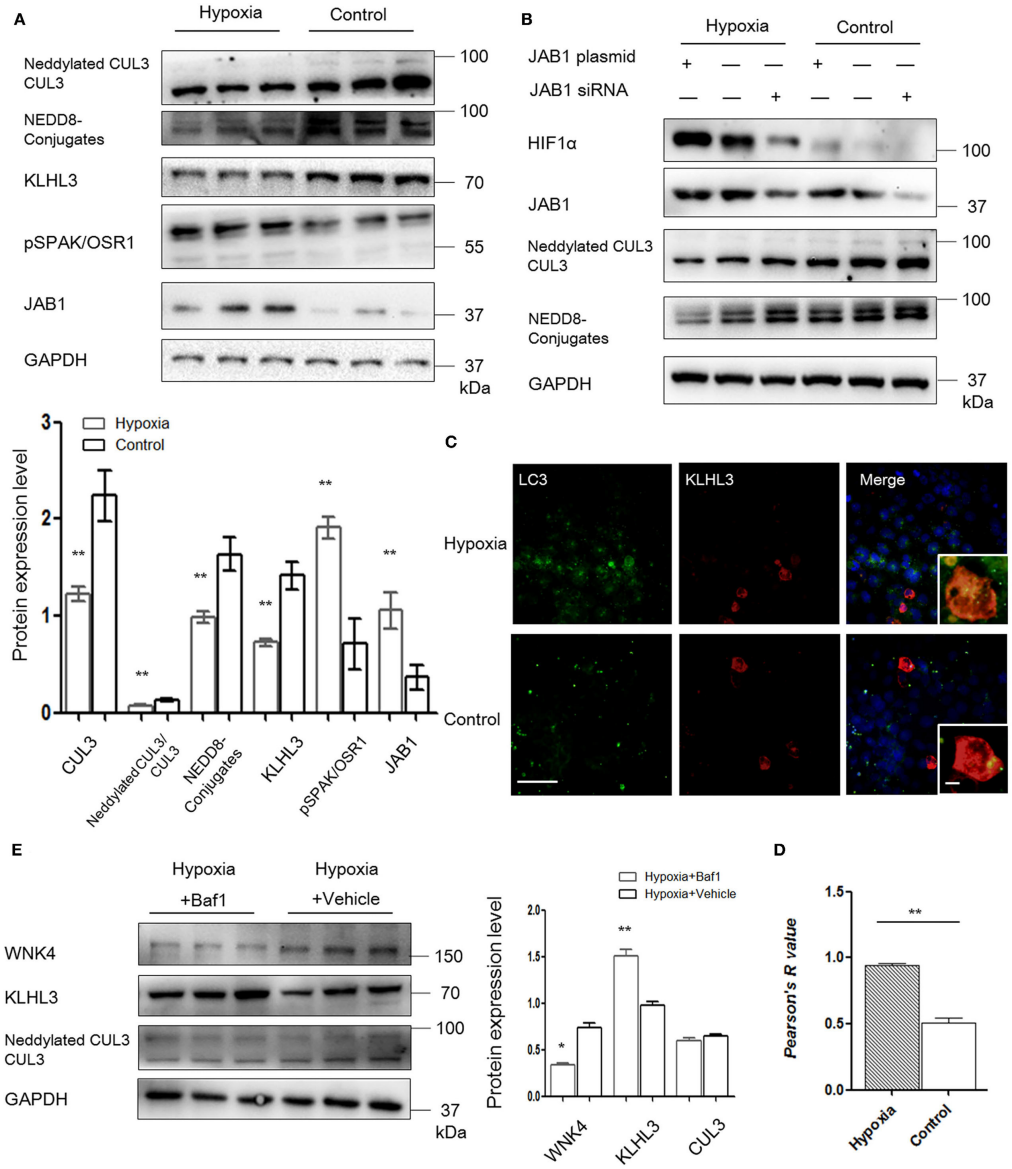
JAB1 promoted HIF1α expression and decreased neddylation of CUL3, and KLHL3 degradation was mediated by autophagy in HEK293 cells under hypoxia. **(A)** Protein levels of CUL3 abundance and neddylation, NEDD8 conjugates, adaptor KLHL3 and downstream phosphorylated SPAK/OSR1, and member of the COP9 signalosome JAB1 were detected in HEK293 cells after transfection with KLHL3. Expressions of these proteins in HEK293 cells were all statistically significant (*t*-test) after normalization to GAPDH between hypoxia (*n* = 3) and control (*n* = 3) group. **(B)** Representative images of protein levels of hypoxia-inducible factor HIF1α, JAB1, CUL3 abundance and neddylation in HEK293 cells after transfected with JAB1 and its siRNA, respectively (*n* = 3). **(C**, **D)** Representative immunofluorescence images of subcellular localization of KLHL3 and autophagy marker LC3. The yellow fluorescence indicated the overlap of KLHL3 and LC3 expression. Scale bars: 100 μm. Statistical analyses of KLHL3 and autophagy marker LC3 colocalization using Pearson correlation coefficient in HEK293 cells (*n* = 10–15). **(E)** Representative immunoblots of WNK4, KLHL3, and CUL3 abundance and neddylation in HEK293 cells after administration with inhibitor of autophagy bafilomycin A1 (Baf1) under hypoxia. Data are presented as the mean ± SEM (*p < 0.05 and **p < 0.01).

Given the evidence that CRL3 was involved in the pathogenesis of PE, we then evaluated the effects of hypoxia on activity of CUL3 and KLHL3/KLHL2 degradation in HEK293 and MOVAS cells. HEK293 cells transfected with KLHL3 were incubated under hypoxia for 16 h. As shown in Figure 5A, KLHL3 expression was significantly decreased in the hypoxia group (*p* < 0.01), and abundance and neddylation of CUL3 were also decreased (*p* < 0.01 and *p* < 0.01, respectively). Similarly to our findings in *in vivo* study, JAB1 expression and pSPAK/OSR1 abundance were significantly increased (*p* < 0.01 and *p* < 0.01, respectively).

To investigate the relationship between JAB1, HIF1α, and CUL3, HEK293 cells were transfected with JAB1, JAB1 siRNA, and a scrambled control, respectively. Cells were then incubated under either 21% O_2_ or 1% O_2_. As shown in Figure 5B, higher JAB1 abundance was associated with higher HIF1α abundance and lower CUL3 neddylation under hypoxia. Elevated JAB1 expression slightly increased HIF1α abundance and significantly decreased the CUL3 neddylation under normoxia.

To further illuminate the underlying mechanism of KLHL3 degradation under hypoxia, we detected the subcellular distribution of transfected KLHL3 and found a significant punctate cytoplasmic pattern of KLHL3. As shown in Figures 5C,D, the overlap of LC3 and KLHL3 in HEK293 cells was significantly increased under hypoxia (*p* < 0.01), suggesting that autophagy contributed to KLHL3 degradation under hypoxic conditions. Autophagy was increased under hypoxia, then HEK293 and MOVAS cells were both administered with autophagy inhibitor Baf1 (bafilomycin A1). As shown in Figure 5E, KLHL3 expression was substantially increased, and WNK4 abundance was decreased after Baf1 administration (*p* < 0.01 and *p* < 0.05, respectively). Abundance and neddylation of CUL3 were not significantly different. Thus, we suggested that Baf1 administration could inhibit the autophagy-mediated degradation of KLHL3 under hypoxia.

### Downregulation of CRL3 Function and Activation of SPAK/OSR1 and PDE5 in MOVAS Cells Under Hypoxia

**Figure 6:**
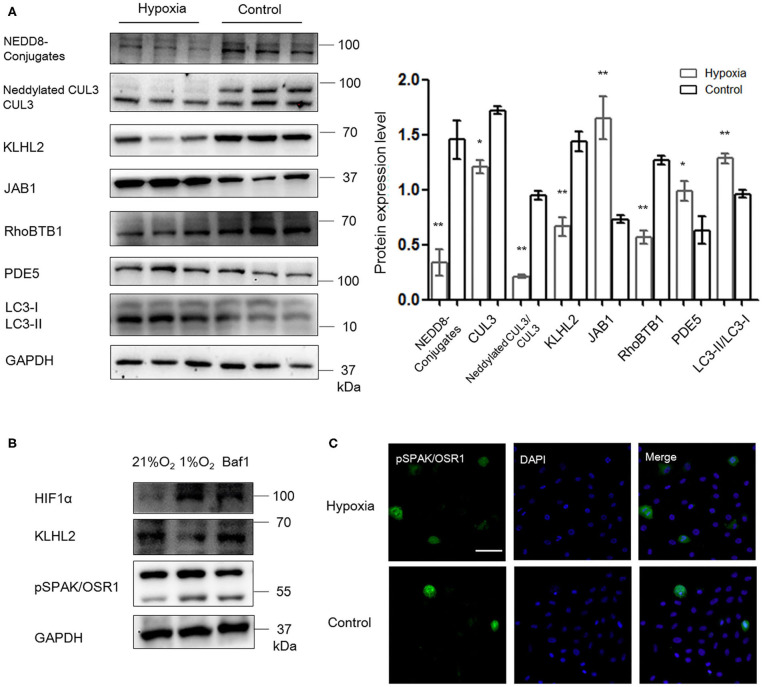
Hypoxia downregulated the function of CRL3 and increased autophagy-mediated degradation of KLHL2 in MOVAS cells. **(A)** Protein levels of NEDD8 conjugates, the components of CRL3, including CUL3 abundance and neddylation, adaptors KLHL2 and RhoBTB1 with substrate PDE5, member of the COP9 signalosome JAB1, and autophagy marker LC3-II/LC3-I were detected in MOVAS cells of hypoxia and control groups. Expressions of these proteins in the MOVAS cells were all statistically significant (*t*-test) after normalization to GAPDH among hypoxia (*n* = 3) and control (*n* = 3) group. **(B)** Representative immunoblots of the adaptor KLHL2, hypoxia-inducible factor HIF1α, and downstream phosphorylated SPAK/OSR1 in MOVAS cells after administration with inhibitor of autophagy bafilomycin A1 (Baf1) under hypoxia (*n* = 3). **(C)** Representative immunofluorescence images of subcellular localization of phosphorylated SPAK/OSR1 in MOVAS cells between the hypoxia and control groups. Scale bars: 100 μm. Data are presented as the mean ± SEM (*p < 0.05 and **p < 0.01).

As shown in Figure 6A, abundance and neddylation of CUL3 were substantially decreased in MOVAS cells under hypoxia (*p* < 0.05 and *p* < 0.01, respectively), and NEDD8-conjugates expression was decreased as well (*p* < 0.01). Meanwhile, JAB1 expression was increased (*p* < 0.01), and KLHL2 and RhoBTB1 protein levels were significantly decreased (*p* < 0.01 and *p* < 0.01, respectively) under hypoxia. As a result, increased pSPAK/OSR1 and PDE5 abundance was detected under hypoxia (*p* < 0.01 and *p* < 0.05, respectively). LC3-II/LC3-I expression was increased in MOVAS cells under hypoxia (*p* < 0.01). Furthermore, Baf1 treatment decreased the degradation of KLHL2 and abundance of pSPAK/OSR1 in MOVAS cells under hypoxia (Figure 6B). Subsequently, we detected the subcellular distribution of endogenous pSPAK/OSR1 in MOVAS cells by IF (Figure 6C). Phosphorylated SPAK/OSR1 signal, which was mainly located in the cytoplasm, substantially increased under hypoxia (*p* < 0.05). These *in vitro* findings demonstrate that under hypoxia/ischemia, the activities of CUL3 and its adaptors were both significantly downregulated.

## Discussion

PE is a leading cause of perinatal maternal death, particularly in developing countries. Our current work demonstrated that the abundance and neddylation of CUL3, as well as the adaptors of CRL3, including RhoBTB1 and KLHL2 were markedly decreased in the pre-eclamptic spiral arteries. As a result, CRL3 substrates, including PDE5, WNK3, and RhoA, were all upregulated. PDE5 accumulation impairs vasorelaxation, and RhoA, cooperating with WNK3 and WNK1, promotes vasoconstriction and induces the proliferation of VSMCs (12, 31). Our study suggests the downregulation of CRL3 function contributes to the abnormal spiral arteries remodeling in PE, which leads to a reduction of uteroplacental blood flow and promotes placental ischemia/hypoxia. Similar changes of CRL3 dysfunction and elevated substrates were found in placentas under the PE condition. As PE remits after delivery, the placenta is deemed responsible (4). CRL3 dysfunction in the placenta impairs vasodilation and causes accumulation of vasoconstrictive substrates and placental ischemia. This results in additional release of circulating bioactive factors including HIF1α into maternal circulation.

HIF1α is the major circulating bioactive factor in PE, and downregulated HIF1α can ameliorate the symptoms including hypertension and proteinuria in PE animal models (32). It was reported that the expression of JAB1, a key component of CSN, was increased in PE placentas (10), and interestingly, JAB1 was shown to interact directly with HIF1α and increased HIF1α protein levels under hypoxia (24). In this study, we verified that both HIF1α and JAB1 abundance was significantly increased in the placentas of PE patients, as well as the aortic tissues and kidney of pregnant l-NAME–treated mice. Our *in vitro* data also suggest that higher JAB1 abundance was associated with higher HIF1α expression. Speculation remains regarding the accumulation of HIF1α protein as a result of the decreased proteasomal activities caused by loss of neddylation of a cullin protein involved in HIF1α degradation.

Under hypoxia, increased HIF1α causes decreased expression of PPARγ, which increases proliferation of distal pulmonary arterial smooth muscle cells and promotes vascular remodeling, resulting in pulmonary hypertension (33). Compared to healthy pregnant women, the level of circulating PPARγ activators was significantly decreased in pre-eclamptic patients (34). Indeed, our findings suggested that PPARγ abundance was significantly decreased in the spiral arteries, placentas of PE patients, and aortic tissues of pregnant l-NAME–treated mice. Moreover, increased medial wall thickness and vascular remodeling were observed in the aortic tissues of the l-NAME group. It was reported that dominant-negative mutations of PPARγ resulted in decreased abundance of CUL3, which explains the possible mechanism of decreased CUL3 in PE revealed in this study (13). CSN directly binds to cullin protein and controls the balance of neddylation and deneddylation. Increased abundance of JAB1, denoting an activated deneddylation state, impaired the neddylation of CUL3 in the vasculature and kidney under PE. Both decreased abundance and decreased neddylation contribute to the inactivation of CUL3. In addition, both RhoBTB1 and KLHL2 abundance was decreased after l-NAME administration, which indicates that adaptors of CUL3 are also downregulated in maternal vasculature. Inactivation of CUL3 and its adaptors, causing decreased proteasomal degradation activities of CRL3, leads to accumulation of PDE5 and WNK kinases and increased RhoA/ROCK activity. These findings in this study are consistent with our hypothesis that dysfunction of CUL3-KLHL2-WNK3/WNK1 and CUL3-RhoBTB1-PDE5 signals disrupts the balance of vasoconstriction and vasodilation and promotes progressive hypertension in PE patients.

Besides the vascular effect discussed above, WNK kinase activation in distal renal tubules also contributes to hypertension in PE. PE animal models showed decreased glomerular filtration rate (GFR) and renal plasma flow (4, 29). Impaired vasodilation and relatively lower GFR demonstrated ischemia of kidneys, which is in accordance with an increased renal HIF1α expression. CUL3-KLHL3-WNK4-NCC signal in the kidney showed similar changes as those in the aortic tissues after l-NAME administration.

It was reported that the decreased abundance of CUL3 shown in heterozygous CUL3 mice alone did not result in any changes of NCC abundance in the kidney (35). However, under PE, CUL3 abundance, and CUL3 neddylation state are decreased, and its specific adaptors expressed in the kidney and vasculature are all downregulated, which accounts for the NCC activation and impaired vasodilation. Our study revealed that the downregulation of CRL3 function affected both the renal tubule sodium absorption and the vascular tone under PE. This explains why PE patients often show severe hypertension and cardiovascular diseases. The length and area of distal tubules expressing NCC were substantially increased in pregnant l-NAME–treated mice, suggesting that DCT remodeling occurs during the development of PE. Vascular and DCT structural remodeling might help to explain why PE patients still have a higher risk of suffering cardiovascular and kidney diseases and later hypertension occurrence even after delivery (36).

Adaptors are responsible for the target selectivity and specificity for ubiquitin degradation by CRLs. They can be degraded by several pathways, including autophagy and proteasomal degradation (9, 28). Under proteasome inhibition, increased autophagy decreased KLHL3 expression (28). Decreased abundance of mTOR and increased LC3-II/LC3-I expression suggest increased autophagy after l-NAME administration. Subcellular distribution supports that the overlap between KLHL3 and LC3 signal was significantly increased under hypoxia, and inhibitor of autophagy increased KLHL3/KLHL2 expression. Thus, we demonstrated that increased autophagy-mediated degradation contributed to the overdegradation of adaptors of CUL3 in PE.

Effective therapy of PE is still lacking, at least partially because of the poor understanding of this disease. PPI administration has been applied to PE patients, and these users showed reduced gestational hypertension (37). However, PPI is not an antihypertensive drug and has no effect on any other type of hypertension. A recent study has indicated that PPI plays a potential role in vasodilation in PE maternal animal model and isolated aortic tissues of PE patients and reduces blood pressure in PE patients (22). Our study suggested that PPI administration partially downregulated HIF1α expression and increased PPARγ abundance and then upregulated abundances of CRL3 components in l-NAME–treated maternal renal tubules and vessels. PPI treatment could partially decrease the accumulation of PDE5, WNK kinases, and RhoA/ROCK activity in l-NAME–treated mice. Our findings highlight a possible mechanism and therapeutic target for treatment of PE. Also, our data further support that downregulation of CRL3 components plays a key role in the pathogenesis and development of PE.

There are some limitations to our study. First, we did not check any other transporters or sodium channels along the renal tubule in this study, and WNK4 was reported to activate the Na–K–Cl cotransporter 2 (NKCC2) in thick ascending limb. Therefore, we cannot exclude other mechanisms that may increase renal sodium absorption (38). Second, telemetry is considered the most reliable source of blood pressure data. Our selection to use a tail-cuff method, a decision based on technical reasons, may have decreased the accuracy of our data.

**Figure 7:**
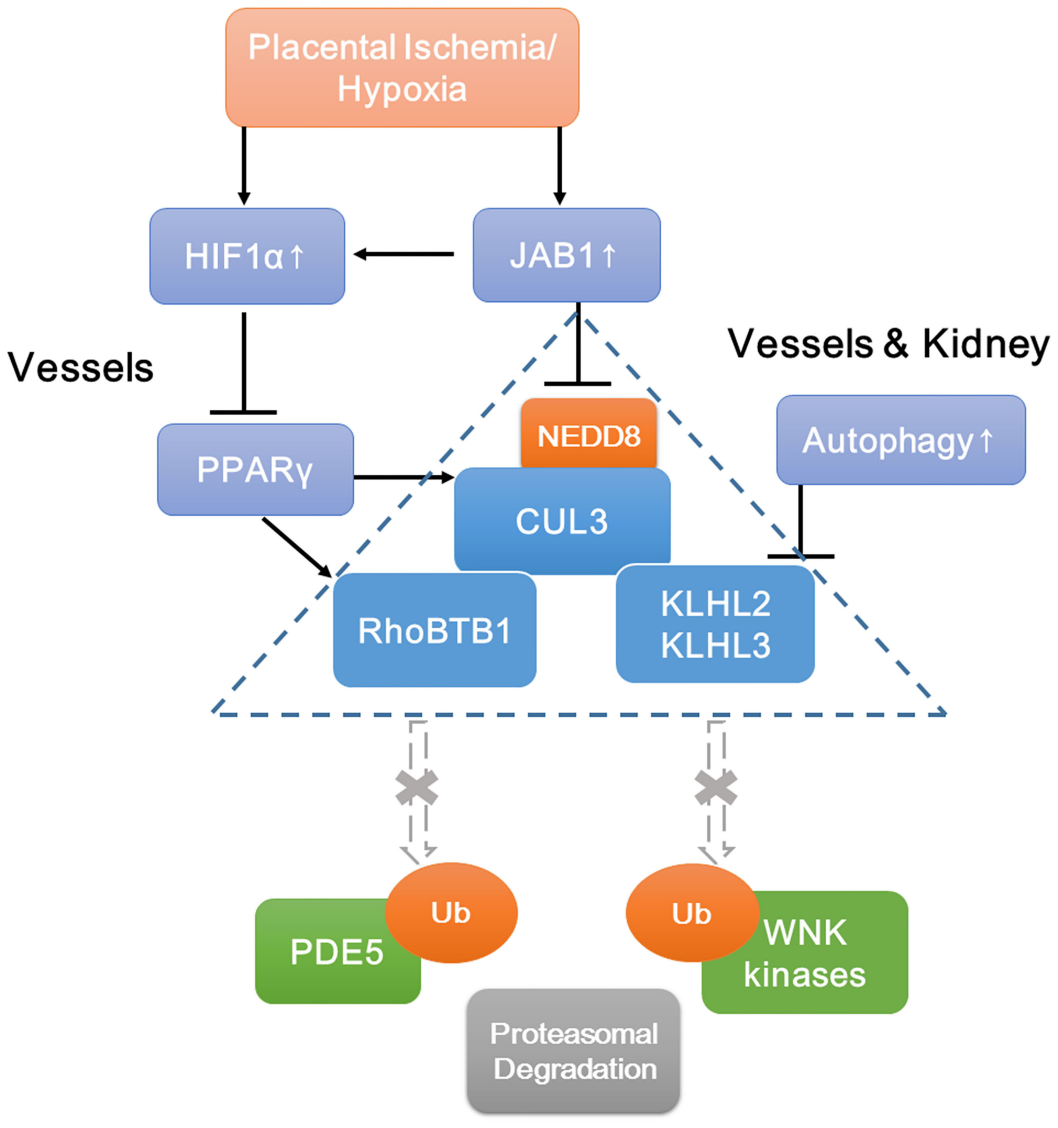
Schematic view of molecular mechanisms of accumulation of WNK kinases and PDE5 under preeclampsia. Increased JAB1 abundance stabilizes HIF1α and upregulates its expression under ischemia/hypoxia. Increased HIF1α decreases PPARγ expression and then downregulates CUL3 abundance. Upregulated CSN also causes lower CUL3 neddylation. Decreased abundance and neddylation of CUL3 lead to CUL3 dysfunction. Increased autophagy promotes degradation of adaptors KLHL3/2, and decreased abundance of PPARγ downregulates adaptor RhoBTB1 expression. Subsequent accumulation of substrates including WNK kinases and PDE5 results in the abnormal vasoconstriction and vasodilation and NCC activation. JAB1, a component of the COP9 signalosome; HIF1α, hypoxia-inducible factor 1α; PPARγ, peroxisome proliferator–activated receptor γ.

Although PE is a very heterogeneous disease, our study suggests that the CRL3 function and abundance are implicated ubiquitously in the pathophysiology of PE, which result in inadequate spiral artery remodeling, poor perfusion of the placenta, and maternal hypertension and renal damage. A schematic view of molecular mechanisms of PE is shown in Figure 7. JAB1 stabilizes HIF1α and upregulates its expression under ischemia/hypoxia. Accumulated HIF1α decreases PPARγ expression and then downregulates the expression of CUL3; meanwhile, upregulated CSN also causes lower CUL3 neddylation, both of which contribute to CUL3 dysfunction. Increased autophagy promotes degradation of KLHL3/2, and decreased PPARγ causes lower abundance of RhoBTB1. The subsequent accumulation of WNK kinases and PDE5 results in the abnormal vascular contraction and vasodilation and NCC activation. Together, these findings demonstrate that CRL3 mediates renal and vascular damages in PE patients, and the components of CRL3 may be potential therapeutic targets against PE.

## Data Availability Statement

The original contributions presented in the study are included in the article/Supplemental Materials, further inquiries can be directed to the corresponding author.

## Ethics Statement

Ethical approval was obtained for this study from the Human Research Ethics Committee of Xin Hua Hospital Affiliated to Shanghai Jiao Tong University School of Medicine. Animal experiments were performed in accordance with the guidelines set by the Animal Care and Use Committee of Xin Hua Hospital Affiliated to Shanghai Jiao Tong University School of Medicine.

## Author Contributions

YZ conceived the study, carried out all the experiments, analyzed the data, and wrote and edited the manuscript. YZ designed and interpreted the results with GJ and CZ. CZ conceived the study, supervised the work, and edited the manuscript. All authors contributed to the article and approved the submitted version.

## Conflict of Interest

The authors declare that the research was conducted in the absence of any commercial or financial relationships that could be construed as a potential conflict of interest.
