# Supplementary material for: Downregulation of Cullin 3 Ligase Signaling Pathways Contributes to Hypertension in Preeclampsia
Source: Front Cardiovasc Med. 2021 Apr 13;8:654254. doi: 10.3389/fcvm.2021.654254 (PMC8076533; doi:10.3389/fcvm.2021.654254)
Supplement: Supplementary file 1 [file Table_1.DOCX]

| **Antibody name** | **Source** | **Dilution** |
| --- | --- | --- |
| anti-KLHL3/KLHL2 antibody | Sigma-Aldrich | WB 1:1000 |
| anti-KLHL3/KLHL2 antibody | Proteintech | WB 1:1000 |
| anti-NEDD8 antibody | Cell Signaling Technology | WB 1:1000 |
| anti-CUL3 antibody | Cell Signaling Technology | WB 1:1000 |
| anti-WNK4 antibody | Ellison lab | WB 1:1000 |
| anti-WNK3 antibody | Ellison lab | WB 1:1000 |
| anti-WNK1 antibody | Abclonal | WB 1:1000 |
| anti-total NCC antibody | Ellison lab | WB 1:1000, IF 1:200 |
| anti-T53 pNCC antibody | Ellison lab | WB 1:1000, IF 1:100 |
| anti-RhoBTB1 antibody | Abclonal | WB 1:1000 |
| anti-PDE5 antibody | Abclonal | WB 1:1000 |
| anti-JAB1 antibody | Santa Cruz | WB 1:1000 |
| anti-pSPAK/OSR1 antibody | Millipore | WB 1:1000, IF 1:200 |
| anti-αSMA antibody | Servicebio | IF 1:200 |
| anti-RhoA antibody | Cell Signaling Technology | WB 1:1000 |
| anti-pMYPT1 antibody | Cell Signaling Technology | WB 1:1000 |
| anti-HIF1α antibody | Cell Signaling Technology | WB 1:1000 |
| anti-PPARγ antibody | Cell Signaling Technology | WB 1:1000 |
| anti-LC3 antibody | Cell Signaling Technology | WB 1:1000, IF 1:100 |
| anti-mTOR antibody | Cell Signaling Technology | WB 1:1000 |
| anti-GAPDH antibody | Cell Signaling Technology | WB 1:1000 |
| anti-HA tag antibody | Cell Signaling Technology | IF 1:100 |

**Table S1**. Antibodies used for Western blot and IF.
