# Supplementary material for: Downregulation of Cullin 3 Ligase Signaling Pathways Contributes to Hypertension in Preeclampsia
Source: Front Cardiovasc Med. 2021 Apr 13;8:654254. doi: 10.3389/fcvm.2021.654254 (PMC8076533; doi:10.3389/fcvm.2021.654254)
Supplement: Supplementary file 2 [file Data_Sheet_1.DOCX]

**Figure S1. Downregulation of CRL3 function with accumulation of WNK kinases, PDE5 and RhoA was detected in the spiral arteries of PE patients.** Western blotting was performed in the spiral arteries of PE patients and healthy pregnancies, including: 1) smooth muscle marker αSMA, 2) CUL3 abundance and neddylation, and the member of the COP9 signalosome JAB1, 3) the adaptors of CRL3 including KLHL2 and RhoBTB1 and their specific substrates including WNK3 and PDE5, and 4) another substrate RhoA and cardioprotective molecule PPARγ. Expressions of these proteins in the spiral arteries were all statistically significant (*t* test) after normalization to GAPDH between PE (n=3) and Control group (n=3). PE indicates preeclampsia and Con indicates Control. Data are presented as the mean ± SEM. **p*<0.05 and ***p*<0.01.

**Figure S2. A-C,** **Thickness of aortic tissues wall and renal vessels wall were both significantly increased in the pregnant L-NAME treated mice.** A, Representative HE staining and Masson’s trichrome staining images of the aortic tissues from Control (n=10), L-NAME (n=10) and L-NAME + PPI group (n=10). Scale bars: 100 μm. B, Histological analysis (ANOVA with Tukey *post hoc* analysis) of wall thickness. Data are presented as the mean ± SEM (**p*<0.05 and ***p*<0.01). C, Representative Masson’s trichrome staining and immunofluorescence staining images of smooth muscle marker αSMA in the renal vessels among Control (n=6), L-NAME (n=6) and L-NAME + PPI group (n=6). Scale bars: 100 μm. **D**, **Representative Real-time qRT-PCR analysis of KLHL2/3 and CUL3 expression.** Six Control and L-NAME aortic tissues/kidney were dissected in one panel, respectively. Data were normalized using the reference gene GAPDH.
