# Supplementary figures and images for: Downregulation of Cullin 3 Ligase Signaling Pathways Contributes to Hypertension in Preeclampsia
Source: Front Cardiovasc Med. 2021 Apr 13;8:654254. doi: 10.3389/fcvm.2021.654254 (PMC8076533; doi:10.3389/fcvm.2021.654254)

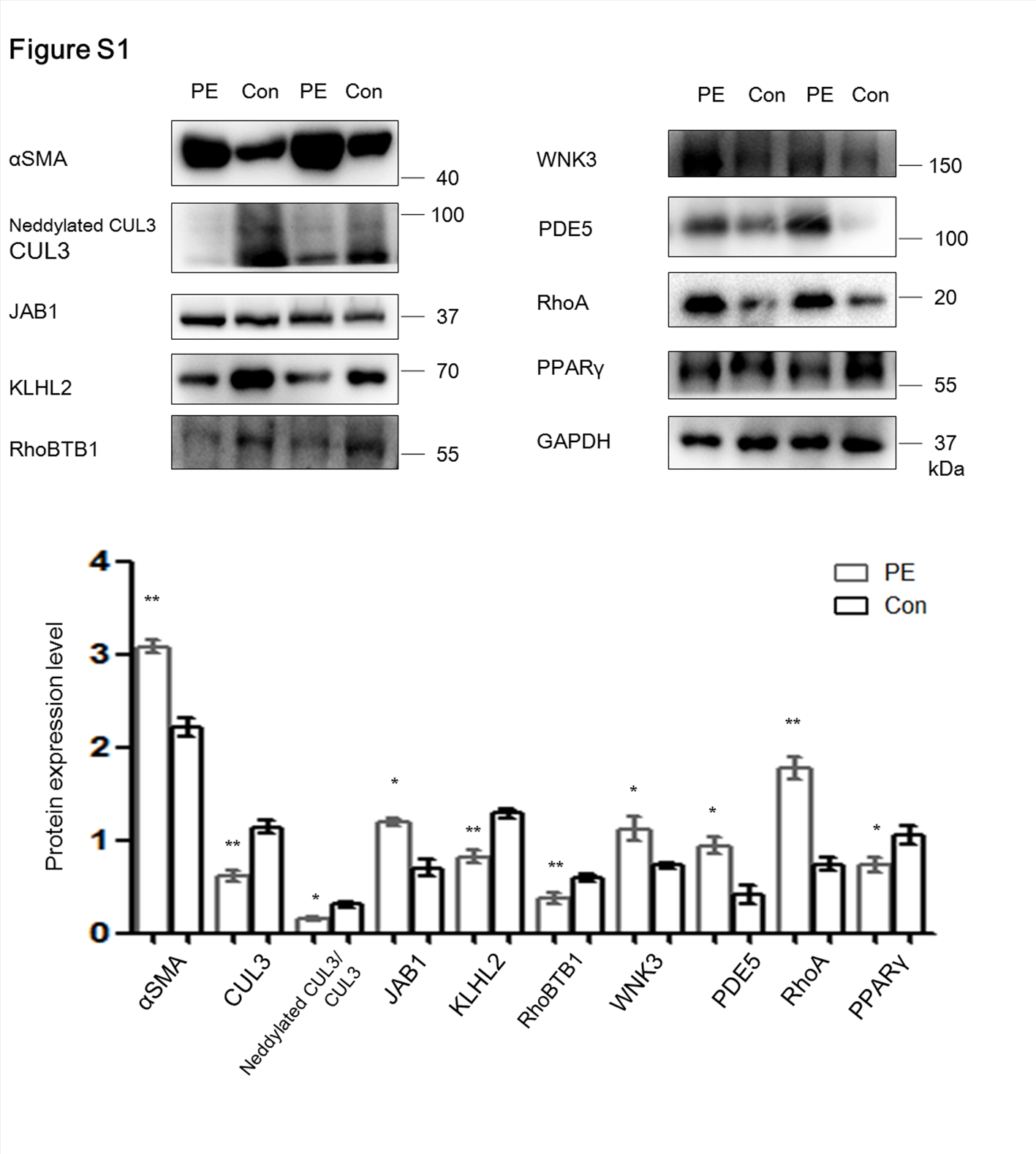

Supplement: Supplementary file 3 [file Image_1.TIF]

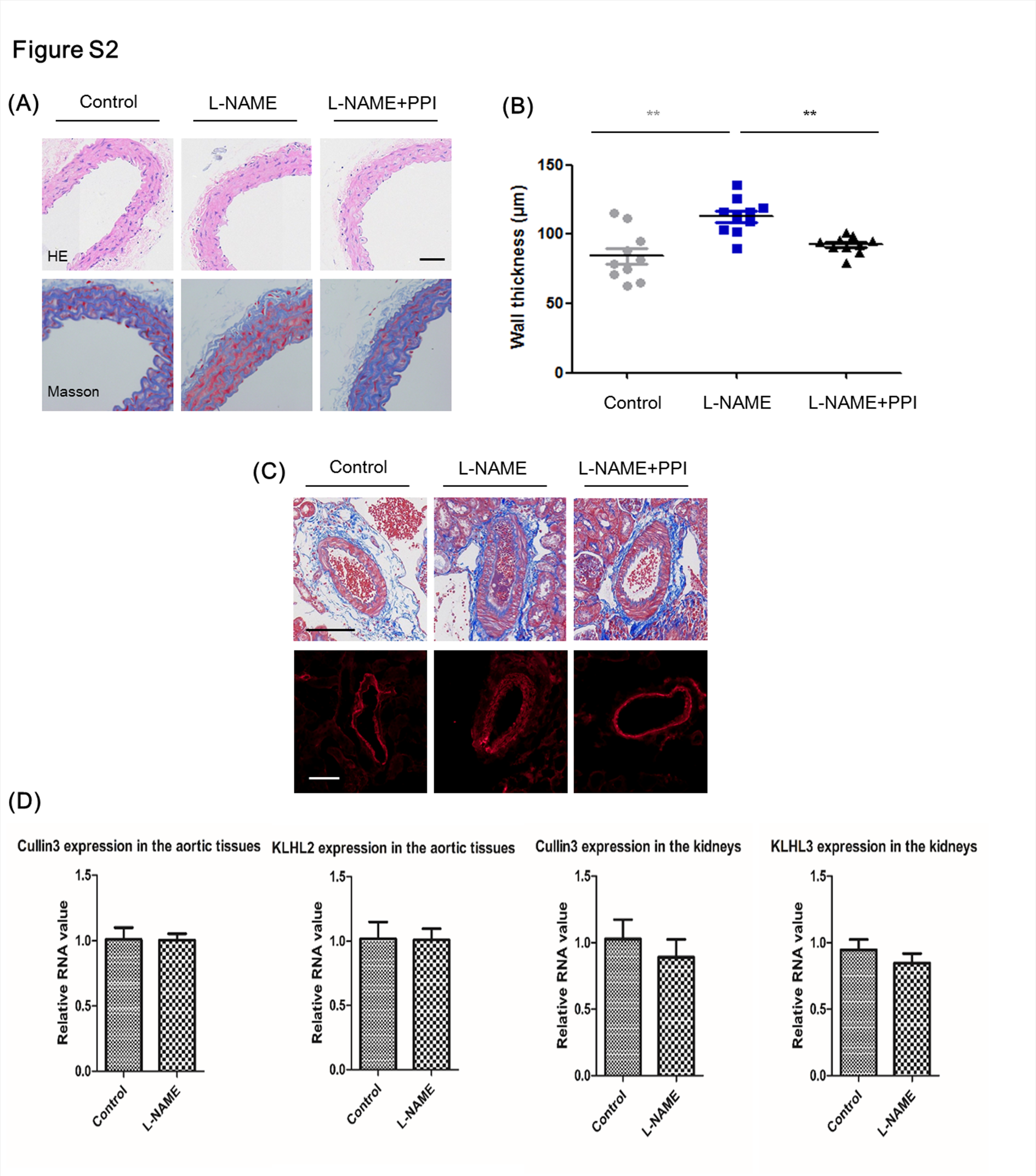

Supplement: Supplementary file 4 [file Image_2.TIF]
